# Long-term predictors of severe exacerbations and mortality in a cohort of well-characterised adults with asthma

**DOI:** 10.1186/s12931-021-01864-z

**Published:** 2021-10-20

**Authors:** Oliver Djurhuus Tupper, Charlotte Suppli Ulrik

**Affiliations:** 1 Respiratory Research Unit, Department of Respiratory Medicine, Hvidovre Hospital, Copenhagen University Hospital-Hvidovre, Kettegaard Alle 30, 2650 Hvidovre, Denmark; 2 Institute of Clinical Medicine, University of Copenhagen, Copenhagen, Denmark

**Keywords:** Asthma, Mortality, Exacerbations, Airway hyperresponsiveness, TRAIL

## Abstract

**Background:**

We aimed to explore long-term predictors of severe exacerbations and mortality in adults with well-characterised asthma.

**Study design and methods:**

Adults (aged ≥ 15) with an objectively verified diagnosis of asthma were recruited from a Danish respiratory outpatient clinic between 1974 and 1990. All individuals were followed in Danish registries for vital status, hospital admissions for asthma and cause of death until end of 2017. Predictors of exacerbations were obtained from a repeated measures model. Standardised mortality rates (SMR) for all-causes were compared with the Danish background population. Hazard ratios for mortality were obtained from a cox proportional hazards model in a two-step process.

**Results:**

At baseline, the cohort comprised 1071 patients (mean age 38, SD 16, 61% women), of whom 357 (33%) died during follow-up, with 93 (26%) dying from asthma (primary diagnosis). We found an SMR of 1.24 (95% CI 1.11–1.37, p < 0.001) for all-cause mortality. Baseline predictors for asthma-related death and repeated severe exacerbations were increasing age, ever smoker, FEV_1_ < 80% pred., high blood eosinophils, longer duration of symptoms and use of SABA > twice daily. Being non-atopic, having a positive histamine challenge test and symptoms more than twice a week were also predictors of repeated exacerbations.

**Conclusions:**

Markers of poor asthma control, including high use of SABA, are predictors of long-term exacerbation rate and mortality over 30 years in patients with well-characterised asthma. Improving asthma control, including lung function and reducing use of reliever medication, is vital for improving the long-term outcome of asthma.

**Supplementary Information:**

The online version contains supplementary material available at 10.1186/s12931-021-01864-z.

## Introduction

Asthma is one of the most common diseases worldwide [1]. Also, asthma places a massive burden due to profound societal and healthcare costs [2, 3].

A multitude of short-term studies, case reviews and national cross-sectional studies have provided us with a good understanding of critical short-term risks for exacerbations and mortality in persons who have asthma. Where age, smoking, poor asthma control, insufficient asthma medication, pulmonary function and lack of follow-up are vital factors [1, 4–6].

Exacerbations present a significant burden on persons with asthma. Exacerbations lead to contact with or admission in the healthcare sector, and in some cases, near-fatal or fatal asthma [7]. Previous studies have found the following factors to be associated with exacerbations,Age, lung function, comorbidities, sputum eosinophils and disease control [8, 9]. However, the majority of these studies have a relatively short follow-up. As the severity for individual asthma patients can vary considerably, and there appears to be substantial instability of phenotypes, potentially factors predictive of short-term outcomes may change long-term [10, 11].

Very few cohort studies have reported risk factors for asthma-related mortality [12, 13]. The majority of information we have is based on all-cause mortality cohorts or older case–control studies [5, 14–16]. Many of the short-term factors also appear to affect long-term all-cause mortality, particularly age, smoking and reduced lung function. However, the long-term consequences and risks for exacerbations and, particularly, asthma-related mortality remain an area ripe for further knowledge.

This long-term Danish asthma cohort study aimed to investigate potential risk factors identified at cohort enrolment for repeated exacerbations, all-cause and asthma-related mortality in adults with well-characterised asthma. Additionally, to investigate the mortality rate in adults with asthma compared with the Danish background population.

## Methods

This was a long-term observational cohort study. The cohort has previously been described in Ulrik et al. [17] and Ali et al. [18] The Cohort consists of consecutive persons age 13 or above, referred to the Allergy and Chest Clinic Frederiksberg Hospital, Denmark, between 1974 and 1990. All persons were referred due to known or suspected asthma. The cohort will be referred to as the treatable traits in asthma—impact on long-term outcome (TRAIL) Cohort.

The study was approved by the ethical committee for the Capital Region of Denmark (H-17025043) and The Danish Data Protection Agency (2013-41-2618).

### Cohort

The cohort comprised all persons diagnosed with and followed for asthma between 1974 and 1990 at Frederiksberg Respiratory and Allergy Clinic, Denmark. The persons included were ≥ 13 years old at baseline. The diagnosis of asthma was made by a specialist in respiratory medicine based on a typical history (wheezing or attacks of breathlessness; chest tightness; cough triggered by exercise, exposure to allergens or irritants or respiratory infections) and at least one of the following:

1. FEV_1_ reversibility > 15% (and an absolute increase of > 150 ml) after a standard dose of short-acting β_2_-agonist (SABA), oral corticosteroid or both (30 mg/day) for 14 days.
2. Diurnal variability in peak expiratory flow (PEF) rate > 20% and absolute variation > 100 l/min.
3. Positive histamine provocation test, with the provocative concentration of histamine that results in a 20% drop in FEV_1_ (PC_20_) ≤ 8 mg/ml.

All cut-off values are based on clinical practice at baseline. At the time of referral to the respiratory outpatient clinic, a comprehensive history was obtained. Total serum IgE was determined by paper radio-immunosorbent test (Pharmacia). A skin prick test with standard aeroallergens was performed. The blood eosinophil count was determined three times for each patient, the most abnormal result was recorded. Forced expiratory volume in 1. second (FEV_1_) and forced vital capacity (FVC) were measured using a dry-wedge bellows spirometer (Vitalograph). The best of three technically acceptable readings were recorded. The tests were repeated 15 min after inhalation of a standard dose of bronchodilator. Reversibility of FEV_1_ was calculated as (FEV_1_ after – FEV_1_ before)/FEV_1_ before). All baseline data were gathered from patient records. All persons went through the same diagnostic algorithm for persons referred for suspected asthma.

### Outcomes

Information on hospital admissions and emergency department visits were obtained from the Danish national patient registry. Hospital and emergency department admissions with the primary diagnoses as the following were defined as asthma exacerbation: acute lower respiratory infection (ICD-8: 480–486 and ICD-10: DJ12-18) or chronic airway disease (ICD-8: 490–493 and ICD-10: DJ40–47). A new admission within 14 days of discharge was counted as the same exacerbation.

All participants were followed from their baseline visit (from 1974 to 1990) and until 31 December 2017. Information about deaths was obtained from the Danish Death Register. Asthma-related death was defined as the cause of death due to acute lower respiratory infection (ICD-8: 480–486 and ICD-10: DJ12-18) or asthma and COPD (ICD-8: 490–493 and ICD-10: DJ40–47).

Mortality in the Danish background population was calculated based on data from Statistics Denmark.

### Statistics

Differences at baseline were compared using student’s t-test, Mann–Whitney U-test and Fisher’s exact test. The indirect standardised mortality rate (SMR) for the TRAIL cohort was calculated in comparison to all-cause mortality in the entire Danish population. Mortality rates were stratified by age (0–45, 46–69 and ≥ 70 years) and year of death (1974–1989, 1990–1999 and 2000–2017) to account for differences in mortality across age groups and time. The following factors, recorded at baseline, potentially being predictors of exacerbation and death were examined: Age, decade of inclusion in the cohort, sex, childhood- or adulthood-onset, duration of symptoms before baseline, FEV1 predicted, FEV1/FVC ratio, blood eosinophils, total IgE, positive or negative skin prick test, baseline asthma medication, airway hyperresponsiveness to inhaled histamine and tobacco exposure. We stratified eosinophils by: < 0.09, in-between or above 0.4 [19]. Cut-offs for β_2_-reversibility and peak flow variability are based on current Danish guidelines. Cut-off used for PC_20_ based on a study by Woolcock et al. [20]. Annualised exacerbation rate was calculated by dividing the number of total exacerbations with years of follow-up.

We used the Cox proportional hazards model with the length of follow-up as the underlying time scale to examine the associations between characteristics obtained at baseline (1974–1990) and death. The proportional hazards assumptions were checked by testing for a non-zero slope in a generalised linear regression of the scaled Schoenfeld residuals on functions of time in continuous variables, and we checked for parallel, non-crossing Kaplan–Meier curves for categorical variables.

There were missing data on the histamine challenge test (25% missing), this was not included in the main analyses, but as a subgroup analysis including only complete cases.

Factors associated with long-term risk of exacerbations were examined by a modified cox proportional hazards model, the Prentice, Williams and Petersen gap-time model [21]. The model allowed us to account for multiple events for each participant. A gap-time model accounts for the first exacerbation affecting the likelihood of the next exacerbation. Repeated exacerbations were capped at three due to a limited number of observations with higher exacerbation frequency.

The effect of each possible factor on exacerbations and death were determined in two steps: first, in a bivariate model, adjusted for length of follow-up. The multivariable models for exacerbation and death were all adjusted for age, smoking and FEV_1_% pred. The remaining variables included in the multivariable models were selected based on statistically significant association with exacerbation or death in the bivariate analyses. Statistical analyses were performed using SAS Enterprise Guide, version 7.15 (SAS Institute Inc. Cary, NC, USA). A two-tailed p-value < 0.05 was considered statistically significant.

## Results

A total of 1071 persons (649 women) with asthma were included in the present analyses, and the median follow-up was 30 years (IQR 26–35 years).

### Exacerbations

A total of 222 (20.6%) persons in the cohort had exacerbations that required hospital admission between baseline and December 31^st^ 2017; of these, 135 (13%) had one, 48 (4.5%) had two and 39 (3.6%) had three or more (up to a maximum of seven). The median time between enrolment into the cohort and first exacerbation was 18.9 (IQR 10.1–28.0 years). The median time between first and second exacerbation was 78 days (IQR 31 days to 0.5 yrs). The median time to third was 33 days (IQR 20 days to 99 days). Patients with three or more exacerbations had more tobacco exposure (50% more pack-years at baseline), almost all (85%) used SABA more than twice daily, and they had a lower level of lung function (Table 1 and Additional file 1: Table S1). Additionally, they had more pronounced airway responsiveness to histamine (lower PC_20_), and a higher number were non-atopic.

**Table 1:** Comparison of baseline characteristics of the TRAIL cohort, between those who experience 0, 1–2 and 3 + exacerbations

	0 (n = 849)	1–2 (n = 182)	3 + (n = 40)	p-value
Sex, n women (%)	506 (60)	114 (63)	29 (73)	0.228
Age	35 (14)	50 (15)	54 (13)	< 0.001
Decade of inclusion				
1974–1979	115 (14)	40 (22)	9 (23)	0.013
1980–1989	641 (76)	131 (72)	27 (68)	
1990	93 (11)	11 (6)	4 (10)	
Adultonset^a^, n (%)	587 (69)	147 (81)	33 (83)	0.002
Ever smoker^b^, n (%)	283 (33)	74 (41)	22 (55)	0.006
Pack-years^c^	9.7 (8.7)	15 (9.5)	16 (15)	< 0.001
Previous severe exacerbation, n (%)	136 (16)	18 (10)	6 (15)	0.100
Daily symptoms, n (%)	271 (32)	98 (54)	29 (73)	< 0.001
Daily use of β_2_-agonist (> 2 puffs), n (%)	399 (47)	120 (66)	32 (80)	< 0.001
High dose ICS or any dose OCS, n (%)	184 (22)	46 (25)	14 (35)	0.096
Lung function				
FEV_1_% pred	86 (17)	74 (21)	61 (22)	< 0.001
FVC % pred	94 (15)	87 (17)	78 (16)	< 0.001
FEV_1_/FVC ratio	78 (71–82)	70 (58–77)	58 (54–73)	< 0.001
BD reversibility, n (%)				
< 12%	130 (16)	25 (15)	2 (5)	0.221
≥ 12%	688 (84)	145 (85)	35 (95)	
AHR^d^	2.4 (1.7–4.5)	2.1 (1.3–4.6)	2.1 (1.00–3.1)	0.072
Peak flow variability, %	22 (15–29)	22 (13–26)	21 (12–25)	0.200
Blood eosinophils	0.34 (0.21–0.57)	0.38 (0.21–0.57)	0.45 (0.23–0.54)	0.534
Total IgE, IU/l	129 (43–345)	93 (35–317)	125 (33–279)	0.165
Negative skin prick test, n (%)	326 (38)	114 (63)	29 (73)	< 0.001

Data are presented as mean (standard deviation) or median interquartile range), unless otherwise stated

*AHR* airway hyperresponsiveness, *BD* bronchodilator, *FEV*_1_ forced expiratory volume in 1 second, *FVC* forced vital capacity, *ICS* inhaled corticosteroids, *IU* International Unit, *OCS* oral corticosteroids

^a^Age ≥ 18 years

^b^Current or ex-smokers

^c^For ever smokers

^d^Missing data on 266

Factors of time appeared well associated with repeated exacerbations increasing age, adult-onset asthma and duration of symptoms before baseline visit (Table 2). Poor asthma control at baseline was also associated with later exacerbations as poor FEV_1_, daily symptoms, and excessive SABA usage showed higher hazard ratios. Finally, high blood eosinophils, more pronounced AHR and being non-atopic were all associated with repeated exacerbations.

**Table 2:** Predictors of repeated asthma exacerbations

	Bivariate model HR (95% CI)	Multivariable model HR (95% CI)
Sex, women	1.17 (0.94–1.46)	–
Age		
15–45	1.00	1.00
46–69	6.00 (4.67–7.71)**	3.56 (2.64–4.81)**
≥ 70	11.2 (7.29–17.2)**	6.30 (3.91–10.1)**
Decade of inclusion		
1974–1979	1.21 (0.76–1.95)	–
1980–1989	1.10 (0.72–1.68)	–
1990	1.00	–
Years since symptom debut	1.02 (1.01–1.03)**	1.02 (1.00–1.03)*
Adult-onset^a^	1.85 (1.41–2.43)**	1.20 (0.81–1.79)
Ever smoker^b^	1.57 (1.19–1.82)**	1.50 (1.20–1.88)*
Pack-years^c^	1.03 (1.02–1.04)**	1.03 (1.01–1.04)*
Previous severe exacerbation	0.68 (0.50–0.94)*	1.27 (0.89–1.82)
Daily symptoms	2.49 (1.98–3.12)**	1.56 (1.22–1.99)*
Daily β_2_-agonist usage, > 2	2.2 (1.74–2.78)**	1.50 (1.13–1.98)*
ICS prescribed at baseline, any dose	1.37 (1.08–1.73)*	0.93 (0.72–1.19)
Lung function		
FEV_1_ pred. < 80%	3.04 (2.41–3.83)**	1.70 (1.29–2.25)*
FEV_1_/FVC ratio, < 70%	2.84 (2.28–3.55) *	1.13 (0.86–1.49)
BD reversibility^d^		
< 12%	–	–
≥ 12%	1.31 (0.96–1.81)	–
AHR, mg/ml		
< 1	1.00	1.90 (0.99–3.67)
≥ 1 to < 2	0.65 (0.44–0.96)*	1.41 (0.72–2.76)
≥ 2 to < 8	0.56 (0.43–0.80)*	1.92 (1.02–3.60)*
≥ 8	1.01 (0.54–1.89)	1.00
Peakflow variability		
< 20%	1.00	–
≥ 20%	0.84 (0.68–1.04)	–
Blood eosinophils, × 10^9^/l		
< 0.09	1.31 (0.84–2.04)	0.98 (0.60–1.59)
≥ 0.09 to ≤ 0.4	1.00	1.00
> 0.4	1.33 (1.08–1.65)*	1.29 (1.03–1.61)*
Total IgE, ≥ 150 IU/l	0.71 (0.57–0.89)*	0.99 (0.77–1.27)
Negative skin prick test, n (%)	0.36 (0.28–0.45)**	1.67 (1.25–2.23)*

Results from bivariate and multivariable cox proportional hazards (PWP) model shown as hazard ratio (95% CI)

*AHR* airway hyperresponsiveness, *BD* bronchodilator, *FEV*_1_ forced expiratory volume in 1 second, *FVC* forced vital capacity, *ICS* inhaled corticosteroids, *IU* International Unit, *OCS* oral corticosteroids

^a^Age ≥ 18 years

^b^Current or ex-smoker

^c^Only ever-smokers

^d^46 did not have data

*p-value < 0.05. **p-value < 0.001. Multivariable model: Wald Chi^2^ = 321 Degrees of freedom = 15. p < 0.0001

### Mortality

During follow-up, 357 (33%) persons died, of whom 93 died of asthma-related causes (Table 3).

**Table 3:** Comparison of causes of death between persons from the TRAIL cohort and the Danish background population from 1974 to 2017, stratified by age groups 0–69 and 70 +

		TRAIL (n = 357)	Denmark (n = 2 113 293)^a^	Relative Risk	95% CI
Asthma, bronchitis, emphysema and pneumonia	0–69	36 (23%)	26,811 (4%)	5.3	4.0–7.1
	70 +	57 (29%)	85,225 (6%)	5.0	4.0–6.2
Cardiovascular causes					
Ischaemic cardiovascular disease	0–69	18 (11%)	80,118 (13%)	0.89	0.6–1.4
	70 +	21 (11%)	298,467 (21%)	0.5	0.4–0.8
Other cardiac cause	0–69	5 (3%)	18,304 (3%)	1.1	0.5–2.6
	70 +	13 (7%)	95,873 (6%)	1.0	0.6–1.7
Malignancy					
Malignant neoplasm	0–69	32 (20%)	181,116 (29%)	0.7	0.5–1.0
	70 +	29 (15%)	274,408 (18%)	0.8	0.6–1.1
Airway neoplasm	0–69	5 (3%)	60,605 (10%)	0.3	0.1–0.8
	70 +	9 (5%)	69,634 (5%)	1.0	0.5–1.8
Other causes	0–69	62 (39%)	338,517 (54%)	0.7	0.6–0–9
	70 +	70 (35%)	664,233 (45%)	0.8	0.7–1.0

^a^Based on 1981–2017 data from statistics Denmark

All-cause mortality, after adjusting for age and year of death, was higher in the TRAIL cohort compared with the Danish background population with a standardised mortality rate (SMR) of 1.24 (95% CI 1.11–1.37, p < 0.001), which equates to an approximately 24% higher mortality rate among patients with asthma. Additional file 1: Table S2 shows mortality data stratified by age and year of death. Further analyses revealed a substantially higher proportion of deaths due to airway related causes among the TRAIL cohort than the Danish population (Table 3). Interestingly, the most pronounced difference in mortality rate was observed in the younger age groups when compared with the general Danish population. Persons aged 45 and below had an SMR of 2.47 (95% CI 1.63–3.31, p < 0.001), and those aged 46 to 69 years had an SMR of 1.56 (95% CI 1.33–1.80, p < 0.001). While those 70 years and above did not differ from the background population (SMR 0.95 95% CI 0.80–1.09, p 0.477).

A comparison of baseline characteristics of persons still alive and those who died during follow-up can be seen in Table 4 for asthma-related death and all-causes in Additional file 2: Table S3. Our analyses showed that those who died of asthma-related causes were older, more often had daily respiratory symptoms and were more likely to have a negative skin prick test, i.e. being non-atopic, at baseline.

**Table 4:** Comparison of baseline characteristics of the TRAIL cohort, between those still alive and those who died of asthma-related causes

	Alive (n = 978)	Dead (n = 93)	p-value
Sex, n women (%)	599 (61)	50 (54)	0.18
Age, yrs	36 (15)	56 (12)	< 0.001
Decade of inclusion			
1974–1979	144 (15)	20 (22)	0.093
1980–1989	731 (75)	68 (73)	
1990	103 (10)	5 (5)	
Years since symptom debut	4 (2–13)	10 (3–25)	< 0.001
Ever smoker^a^, n (%)	331 (33.8)	48 (52)	< 0.001
Pack-years^b^	7.7 (3.5–14)	15 (7.5–22.3)	< 0.001
History of asthma exacerbation, n (%)	159 (16)	14 (15)	0.883
Daily symptoms, n (%)	340 (35)	58 (62)	< 0.001
Daily β_2_-agonist usage	2 (2–4)	4 (3–6)	< 0.001
ICS dosage at baseline	200 (0–400)	400 (0–600)	0.082
Lung function			
FEV_1_% pred	85 (17.6)	62 (21.5)	0.006
FVC % pred	94 (15)	80 (19)	< 0.001
FEV_1_/FVC ratio	75 (11)	61 (13)	0.024
BD Reversibility, %	18 (16–26)	28 (16–43)	< 0.001
AHR	2.35 (1.60–4.36)	2.00 (1.10–3.98)	0.280
Peak flow variability, %	22 (15–29)	23 (13–26)	0.725
Blood Eosinophils, × 10^9^/l	0.34 (0.21–0.57)	0.42 (0.21–0.57)	0.521
Total IgE, IU/l	125 (41–342)	105 (36–316)	0.317
Negative skin prick test, n (%)	403 (41)	66 (71)	< 0.001

Data are presented as mean (standard deviation) or median interquartile range), unless otherwise stated

*AHR* airway hyperresponsiveness, *BD* bronchodilator, *FEV*_1_ forced expiratory volume in 1 Second, *FVC* forced vital capacity, *ICS* inhaled corticosteroids, *IU* International Units

^a^Current or ex-smokers

^b^For ever smokers

The results from the Cox proportional hazards models are presented in Table 5 and Additional file 2: Table S4 for asthma-related and all-cause mortality, respectively. Age and smoking were associated with both all-cause and asthma-related mortality. A longer time from the onset of symptoms until a person was first seen in the outpatient clinic was associated with a higher HR of asthma-related mortality. Markers of poor asthma control were also associated with long-term mortality risk. High blood eosinophils (> 0.4 × 10^9^/l) were associated with asthma-related mortality, but not all-cause mortality. Additionally, an interaction between asthma onset and blood eosinophils meant the risk for mortality was more pronounced for persons with childhood-onset asthma. The decade of inclusion was associated with a higher risk of all-cause mortality, in that patients with earlier inclusion into the cohort had a higher risk of early mortality. There was no association between inclusion decade and asthma-related mortality or exacerbation risk.

**Table 5:** Predictors of asthma-related mortality

	Bivariate model HR (95% CI)	Multivariable HR (95% CI)
Sex, women	1.33 (0.89–2.01)	–
Age		
15–45	1.00	1.00
46–69	16.4 (9.62–27.9)**	5.11 (2.77–9.43)**
≥ 70	29.0 (12.6–66.5)**	9.00 (3.55–22.9)**
Decade of inclusion		
1974–1979	2.25 (0.84–6.06)	–
1980–1989	1.67 (0.67–4.12)	–
1990	1.00	–
Years since symptom debut	1.05 (1.04–1.06)**	1.05 (1.03–1.07)**
Adult-onset^a^	2.63 (1.49–4.65)**	3.20 (1.42–7.21)*
Ever smoker^b^	2.07 (1.38–3.11)**	2.43 (1.58–3.74)**
Pack-years, only ever smokers	1.06 (1.04–1.08)**	1.03 (1.00–1.07)*
Previous severe exacerbation	0.58 (0.30–1.11)	–
Daily symptoms	3.21 (2.11–4.89)**	1.35 (0.86–2.12)
Daily β_2_-agonist use, > 2 puffs	4.21 (2.57–6.89)**	2.11 (1.26–3.663)*
ICS prescribed at baseline, any dose	1.82 (1.17–2.84)*	1.16 (0.73–1.73)
Lung function		
FEV_1_% pred. < 80%	8.28 (5.04–13.6)**	3.46 (1.94–6.16)**
FEV_1_/FVC ratio, < 70%	6.58 (4.27–10.1)**	1.40 (0.84–2.33)
BD reversibility		
< 12%	1.00	–
≥ 12%	1.70 (0.88–3.29)	–
AHR, mg/ml		
< 1	0.47 (0.17–1.31)	0.95 (0.31–2.94)
≥ 1 to < 2	0.36 (0.13–0.97)*	1.03 (0.34–3.10)
≥ 2 to < 8	0.23 (0.09–0.58)*	1.61 (0.56–4.60)
≥ 8	1.00	1.00
Peak flow variability	0.84 (0.55–1.29)	–
Blood eosinophils, × 10^9^/l		
< 0.09	1.78 (0.79–3.96)	1.23 (0.54–2.81)
≥ 0.09 to ≤ 0.4	1.00	1.00
> 0.4	1.56 (1.02–2.37)**	1.63 (1.05–2.53)*
Total IgE, < 150 IU/L	0.75 (0.49–1.45)	–
Negative skin prick test, n (%)	4.10 (2.62–6.44)**	0.65 (0.40–1.07)

Results from bivariate and multivariable cox proportional hazards model shown as hazard ratio (95% CI)

*AHR* airway hyperresponsiveness, *BD* bronchodilator, *FEV*_1_ forced expiratory volume in 1 second, *FVC* forced vital capacity, *IU* international units, *ICS* inhaled corticosteroids, *OCS* oral corticosteroids

^a^Age ≥ 18 years

^b^Current or ex-smokers.

*p-value < 0.05. **p-value < 0.001. Multivariable model: Wald Chi^2^ = 190 Degrees of freedom = 13. p < 0.0001

For all-cause mortality, an interaction between skin prick test and blood eosinophils, meaning a positive skin prick test was associated with higher all-cause mortality if blood eosinophils were < 0.09 × 10^9^/l.

## Discussion

This was a long-term cohort follow-up of 1071 persons with a doctor’s diagnosis of asthma based on objective criteria followed from baseline between 1974 and 1990 until the end of 2017 or death, whichever came first. Compared with the general Danish population, we found a higher mortality rate among the cohort, primarily in those of younger age. Predictors of asthma-related mortality were older age, longer symptom duration, smoking, reduced lung function, high SABA use, adult-onset disease and high blood eosinophils. Exacerbations were associated with no atopy, daily symptoms and AHR, in addition to the factors associated with mortality.

The average time between exacerbations decreased after each subsequent exacerbation. This finding correlates well with previous studies showing that recent exacerbations increase the likelihood of subsequent exacerbations [22].

Previous exacerbations before baseline were not associated with subsequent exacerbations following baseline. The explanation is likely that previous exacerbations could be any length of time before baseline in the TRAIL study, and as shown previously, the predictive value of previous exacerbations dissipates over 5 years [23].

The effect of increasing age seems to be the most critical predictor of repeated exacerbations across a prolonged period. This effect is not wholly understood but could partially stem from frequent viral respiratory tract infections together with decreased immune cell function. Additionally, a decreased effect of β_2_-adrenergic medicine and increased non-reversible airway obstruction could play a role [24, 25].

Reduced lung function has time and again been proven to predict future exacerbations across the short term [1]. The TRAIL cohort supports the importance of low FEV_1_ as a predictor and shows that its importance prevails across 30 years.

Increased inflammation and corticosteroid insensitivity short-term, followed by long-term airway remodelling, is potentially the cause of smoking’s long-term predictive value of repeated exacerbations [26].

Poor asthma control with daily symptoms or excessive use of reliever medication were of a particularly high risk of long-term exacerbations. As shown by previous studies, excessive use of SABA and poor symptom control leads to hospitalisation and, in the worst case, fatal asthma [27, 28].

To our knowledge, the finding that a negative skin prick test is predictive of repeated exacerbations has not been presented previously. Though when seen as a marker of non-atopy, it is consistent with studies showing that non-atopic asthma is harder to control and therefore have a higher likelihood of exacerbations [29]. There is a clear consensus that high eosinophil count is associated with short-term exacerbation risk, with both population and cohorts studies showing this association [30, 31]. Based on our findings across 30 years, it appears that this risk continues long-term.

We found a substantially higher mortality rate among individuals with asthma compared with the Danish background population, which is well in line with other asthma cohort studies and epidemiological studies [16, 32]. The higher mortality in the TRAIL cohort is in large part due to deaths being asthma-related (Table 1), with 26% of the fatal cases, but also higher a higher all-cause mortality rate among those aged 15–45. This is higher than other studies, Okayama et al. [12] and Lemmetyinen et al. [16] found that respiratory-related deaths accounted for 14% and 1% of deaths, respectively. This could be due to the participants in the TRAIL cohort were followed at secondary care facilities and therefore had more severe asthma than the other two cohorts, which were general population cohorts.

The drop in SMR between 1974 and 1999 and into the early 2000s match that reported by Ebmeier et al. [33]. Yet, despite this drop, mortality remains higher for individuals with asthma. Suggesting that there is still room for improvement, so which factors should we consider when evaluating patients with asthma?

Of the investigated significant factors, we found excessive use of SABA, and high-blood eosinophil count was unique to asthma-related mortality, while the remaining factors were like that of all-cause mortality. Another study found a similar semblance of factors when comparing all-cause and respiratory-related mortality [12]. Potentially treatable traits associated with asthma-related mortality were excessive use of SABA, high blood eosinophils, and low FEV_1_. All have been shown numerous times to affect short-term and all-cause mortality and now, based on our findings, also on long-term asthma-related mortality [5, 15].

Adult-onset and longer duration of symptoms were both associated with an increased risk of asthma-related mortality, also after adjusting for age. This finding is well in line with findings that adult-onset has a poorer prognosis and suboptimal response to treatment than childhood-onset asthma [34]. Longer disease duration has been associated with continuing inflammation and, therefore airway remodelling, which in turn leads to adverse outcomes [35].

In the TRAIL cohort, a higher risk of all-cause mortality was associated with negative skin prick test, and this effect was more pronounced for individuals with low blood eosinophils. This finding is well in line with previous findings that individuals with non-Th2 asthma have poor outcomes [36].

On the other end of the spectrum, individuals with a very high blood eosinophil count have a higher risk of asthma-related mortality, particularly individuals with childhood-onset asthma. This finding goes well in hand with high eosinophils predicting exacerbations as discussed above, though the association with mortality has only rarely been examined previously [37]. Furthermore, this upper limit has been shown to be associated with accelerated lung function decline previously [38].

While inclusion spanned three decades, most patients (54%) were included between 1985 and 1990. We can, therefore, not accurately comment on whether changes in practice from the 70’s to the 90’s affect mortality or exacerbation, as most participants were most likely managed by similar practices.

This study’s unique strength lies in the exceptionally long follow-up and reporting on asthma-related mortality. Not to be overlooked is the large number of participants who all had well-established and well-characterised asthma at baseline. In combination, this provides substantial insight into the path for adult persons followed at outpatient clinics and allows us as practitioners to provide better information at the first visit.

There are a few limitations worth mentioning. We did not gather information on prescribed medicine and symptoms after baseline, as these have most assuredly changed, we cannot account for the effects these changes might have on the disease trajectories of the participants.

All participants were diagnosed with asthma based on objective criteria, and 65% were life-long never-smokers at baseline, and we are therefore confident of this diagnosis at baseline. However, as there were no follow-up visits, we cannot account for whether any participants developed concomitant COPD during the study and can therefore not account for this factor.

All participants were referred to a secondary care clinic, this may limit the generalisability.

The SMR has previously been shown to underestimate the actual mortality rate in comparison with a control group [39]. Meaning the actual mortality rate may be relatively higher than what we have reported.

Numbers of exacerbations is based on admission coding alone and not journal review. There is, therefore, a risk of both under- and overestimating the actual number of severe exacerbations.

Persons with asthma have a higher rate of mortality than the background population. Markers of poor asthma control are associated with both repeated exacerbations and asthma-related mortality. Likewise, adult-onset of asthma was associated with asthma-related mortality and longer time since disease debut was associated with both mortality and exacerbations. Finally, persons with non-atopic non-eosinophilic phenotype are at risk of all-cause mortality, while persons with very high blood eosinophils count are at particularly high risk of exacerbations and asthma-related mortality.

## Supplementary Information

**Additional file 1: Table S1.** Comparison of baseline characteristics of the TRAIL cohort, stratified by annualised exacerbation rate. **Table S2.** Number of deaths by all causes in the TRAIL cohort and the Danish general population, stratified by age at time of death and year of death

**Additional file 2: Table S3.** Comparison of baseline characteristics of the TRAIL cohort, between those still alive and those who died of all causes. **Table S4.** Predictors of all-cause mortality, findings from bivariate and multivariable cox proportional hazards model shown as hazard ratio (95% CI).

## Data Availability

The data are available upon reasonable request, but analysis may require approval from the regional data safety committee for the capital region of Denmark (Videnscenter for dataanmeldelser).

## Abbreviations

AHR: Airway hyperresponsiveness
FEV_1_: Forced expiratory volume in 1. second
FVC: Forced vital capacity
PC_20_: The provocative concentration of histamine that results in a 20% drop in FEV_1_
SABA: Short-acting β_2_-agonist
SMR: Standardised mortality rate
TRAIL: Treatable traits in asthma, the impact on long-term outcome cohort
