# Supplementary material for: Long-term predictors of severe exacerbations and mortality in a cohort of well-characterised adults with asthma
Source: Respir Res. 2021 Oct 20;22:269. doi: 10.1186/s12931-021-01864-z (PMC8529759; doi:10.1186/s12931-021-01864-z)
Supplement: Supplementary file 1 — Additional file 1: Table S1. Comparison of baseline characteristics of the TRAIL cohort, stratified by annualised exacerbation rate. Table S2. Number of deaths by all causes in the TRAIL cohort and the Danish general population, stratified by age at time of death and year of death [file 12931_2021_1864_MOESM1_ESM.docx]

e-table 1 – Comparison of baseline characteristics of the TRAIL cohort, stratified by annualised exacerbation rate.

|  | | | 0 (n =849) | <0.1 (n=134) | ≥ 0.1 (n=88) | p-value |
| --- | --- | --- | --- | --- | --- | --- |
| Sex, n women (%) | | | 506 (60%) | 92 (69%) | 51 (58%) | 0.117 |
| Age | | | 34.9 (14) | 46.3 (15) | 56.5 (13) | <0.001 |
| Adul-tonset^a^, n (%) | | | 587 (69%) | 105 (78%) | 75 (85%) | <0.001 |
| Ever smoker^b^, n (%) | | | 283 (33%) | 54 (40%) | 42 (48%) | <0.001 |
| Pack-years^c^ | | | 9.7 (8.7) | 13.0 (8.5) | 17.7 (13) | <0.001 |
| Previous severe exacerbation, n (%) | | | 136 (16%) | 17 (13%) | 7 (8.0%) | 0.089 |
| Daily symptoms, n (%) | | | 273 (32%) | 65 (49%) | 62 (70%) | <0.001 |
| Daily use of β_2_-agonist (>2 puffs), n (%) | | | 399 (47%) | 82 (61%) | 70 (80%) | <0.001 |
| High dose ICS or any dose OCS, n (%) | | | 184 (22%) | 33 (25%) | 27 (31%) | 0.135 |
| Lung Function | FEV_1_ % pred | | 86 (17) | 79 (19) | 61 (22) | <0.001 |
|  | FVC % pred | | 94 (15) | 90 (16) | 79 (17) | <0.001 |
|  | FEV_1_/FVC ratio | | 78 (71—82) | 73 (63—78) | 58 (49—72) | <0.001 |
|  | BD Reversibility,  n (%) | < 12% | 130 (16) | 19 (15) | 8 (10%) | 0.413 |
|  |  | ≥ 12% | 688 (84%) | 109 (85%) | 71 (90%) |  |
|  | AHR^e^ | | 2.4 (1.7—4.5) | 2.3 (1.3—4.6) | 2.1 (1.0—3.5) | 0.088 |
|  | Peak flow variability, % | | 22 (15—29) | 22 (14—26) | 21 (12—27) | 0.218 |
| Blood Eosinophils | | | 0.34 (0.21—0.57) | 0.34 (0.19—0.52) | 0.48 (0.26—0.61) | 0.024 |
| Total IgE, IU/l | | | 129 (43—345) | 102 (39—317) | 64 (33—299) | 0.103 |
| Negative skin prick test, n (%) | | | 326 (38%) | 75 (56%) | 68 (77%) | <0.001 |

Annualised exacerbation rate = number of exacerbations divided by years of follow-up. Data are presented as mean (standard deviation) or median interquartile range), unless otherwise stated. AHR=Airway Hyperresponsiveness. BD=bronchodilator. FEV_1_= Forced expiratory volume in 1. Second. FVC=Forced vital capacity. ICS=Inhaled corticosteroids. IU=International unit. OCS=Oral corticosteroids. a=age ≥18 years. b=current or ex-smokers. c=For ever smokers. d=Any prescribed dosage. e= missing data on 266.

| Age | Year | TRAIL cohort deaths | Background population deaths | TRAIL Population-time (years) | Background population Population-time (years) |
| --- | --- | --- | --- | --- | --- |
| 0—45 | 1974—1989 | 11 | 58491 | 3664 | 53169784 |
|  | 1990—1999 | 14 | 32799 | 6212 | 32491367 |
|  | 2000—2017 | 8 | 37017 | 4823 | 58024233 |
| 46—69 | 1974—1989 | 38 | 252982 | 1475 | 20523377 |
|  | 1990—1999 | 53 | 147310 | 2929 | 13984446 |
|  | 2000—2017 | 77 | 232701 | 7534 | 29996763 |
| 70+ | 1974—1989 | 10 | 578259 | 1932.5 | 7956131 |
|  | 1990—1999 | 48 | 426826 | 780 | 5695750 |
|  | 2000—2017 | 108 | 715790 | 1606 | 11191378 |

e-table 2 – Number of deaths by all causes in the TRAIL cohort and the Danish general population, stratified by age at time of death and year of death.
