# Supplementary material for: Long-term predictors of severe exacerbations and mortality in a cohort of well-characterised adults with asthma
Source: Respir Res. 2021 Oct 20;22:269. doi: 10.1186/s12931-021-01864-z (PMC8529759; doi:10.1186/s12931-021-01864-z)
Supplement: Supplementary file 2 — Additional file 2: Table S3. Comparison of baseline characteristics of the TRAIL cohort, between those still alive and those who died of all causes. Table S4. Predictors of all-cause mortality, findings from bivariate and multivariable cox proportional hazards model shown as hazard ratio (95% CI). [file 12931_2021_1864_MOESM2_ESM.docx]

Supplemental tables

e-Table 3 Comparison of baseline characteristics of the TRAIL cohort, between those still alive and those who died of all causes.

|  | | | Alive (n=714) | Dead (n=357) | p-value |
| --- | --- | --- | --- | --- | --- |
| Sex, women n (%) | | | 435 (61) | 214 (60) | 0.791 |
| Age | | | 31 (10.5) | 53 (14) | <0.001 |
| Decade of inclusion | | 1974-1979 | 77 (11) | 87 (24) | <0.001 |
|  |  | 1980-1989 | 548 (77) | 251 (70) |  |
|  |  | 1990 | 89 (12) | 19 (5) |  |
| Years since symptom debut | | | 4 (1—11) | 7 (2—20) | <0.001 |
| Adult-onset^a^, n (%) | | | 458 (64) | 309 (86) | <0.001 |
| Ever smoker^b^, n (%) | | | 233 (33) | 146 (41) | 0.008 |
| Pack-years^c^ | | | 6 (3—11) | 14 (8—21) | <0.001 |
| History of asthma exacerbation, n (%) | | | 128 (18) | 45 (13) | 0.028 |
| Daily symptoms, n (%) | | | 218 (31) | 180 (50) | <0.001 |
| Daily doses of rescue β_2_-agonist | | | 2 (2—4) | 3 (2—5) | <0.001 |
| ICS dose prescribed | | | 200 (0—400) | 400 (0—600) | 0.012 |
| Lung Function | FEV_1_ % pred | | 89 (14.7) | 73 (22.3) | <0.001 |
|  | FEV_1_/FVC ratio | | 78 (9.0) | 67 (13.2) | <0.001 |
|  | FEV_1_ bronchodilator reversibility^d^, % | | 18 (15—23) | 21 (16—37) | <0.001 |
|  | AHR^e^ | | 2.35 (1.7—4.5) | 2.3 (1.3—4.6) | 0.360 |
|  | Peak flow variability, % | | 23 (15—29) | 21 (14—29) | 0.275 |
| Blood eosinophils, x10^9^/l | | | 0.34 (0.21—0.57) | 0.38 (0.21—0.56) | 0.540 |
| Total IgE, IU/l | | | 135 (44—350) | 101 (34—320) | <0.05 |
| Negative skin prick test, n (%) | | | 233 (33) | 236 (66) | <0.001 |

Data are presented as mean (standard deviation) or median interquartile range), unless otherwise stated. AHR=Airway Hyperresponsiveness. BD=bronchodilator. FEV_1_= Forced expiratory volume in 1. Second. FVC=Forced vital capacity. IU=International units. a=age ≥18 years. b=current or ex-smokers. c=For ever smokers. d=46 did not have data e= missing data on 266.

e-Table 4 – Predictors of all-cause mortality, findings from bivariate and multivariable cox proportional hazards model shown as hazard ratio (95% CI).

|  | | | Bivariate model  HR (95% CI) | Multivariable model  HR (95% CI) |
| --- | --- | --- | --- | --- |
| Sex, women | | | 1.04 (0.84—1.29) | - |
| Age at baseline | | 15—45 | 1.00 | 1.00 |
|  |  | 46—69 | 9.18 (7.24—11.6)^**^ | 4.89 (3.61—6.61)^**^ |
|  |  | ≥70 | 24.2 (16.5—35.6)^**^ | 11.8 (7.55—18.5)^**^ |
| Inclusion by decade | | 1974—1979 | 2.61 (1.58—4.31)** | 1.92 (1.08—3.39)* |
|  |  | 1980—1989 | 1.67 (1.05—2.67)* | 1.67 (1.02—2.74)* |
|  |  | 1990+ | 1.00 | 1.0 |
| Years since symptom debut | | | 1.03 (1.02—1.04)^**^ | 1.03 (1.02—1.04)^**^ |
| Adult-onset | | | 3.04 (2.34—4.12)^**^ | 2.15 (1.41—3.28)^**^ |
| Ever smoker^a^ | | | 1.36 (1.10—1.67)^**^ | 1.53 (1.21—1.92)^**^ |
| Pack-years^b^ | | | 1.06 (1.05—1.07)^**^ | 1.05 (1.03—1.07)^**^ |
| Previous severe exacerbation^c^ | | | 0.53 (0.37—0.75)^**^ | 1.01 (0.68—1.49) |
| Daily symptoms | | | 1.99 (1.62—2.45)^**^ | 1.20 (0.94—1.53) |
| Daily β_2_-agonist usage, > 2 puffs | | | 1.95 (1.57—2.42)^**^ | 1.13 (0.88—1.45) |
| ICS prescribed at baseline, any dose | | | 1.45 (1.14—1.84)^*^ | 1.08 (0.83—1.4) |
| Lung Function | FEV_1_ % pred, < 80% | | 3.60 (2.91—4.45)^**^ | 1.55 (1.17—2.01)^*^ |
|  | FEV_1_/FVC ratio, <70% | | 3.79 (3.07—4.67)^**^ | 1.25 (0.94—1.66) |
|  | FEV_1_ Reversibility^d^ | < 12% | 1.00 | 1.00 |
|  |  | ≥12% | 1.45 (1.04—2.00)^*^ | 0.81 (0.57—1.16) |
|  | AHR^e^ | | 1.05 (1.02—1.07)^**^ | 1.00 (0.98—1.02) |
|  | Peak flow variability | | 0.88 (0.70—1.10) | - |
| Blood eosinophils, x10^9^/l | | <0.09 | 1.84 (1.25—2.72)^*^ | 1.42 (0.94—2.13) |
|  |  | ≥0.9 to ≤0.4 | 1.00 | 1.00 |
|  |  | >0.4 | 1.29 (1.04—1.60)^*^ | 1.11 (0.88—1.40) |
| Total IgE, IU/l | | | 0.81 (0.65—1.00)^*^ | 1.20 (0.94—1.53) |
| Negative skin prick test, n (%) | | | 3.32 (2.67—4.15)^**^ | 1.92 (1.43—2.56)^**^ |

AHR=Airway Hyperresponsiveness. BD=bronchodilator. FEV1= Forced expiratory volume in 1. Second. FVC=Forced vital capacity. IU=International units. ICS=Inhaled corticosteroids. OCS=Oral corticosteroids. a= current and ex-smokers. b=Includes only ever smokers (n=379). c=exacerbation requiring hospital admission. d=46 did not have data e= missing data on 266. *=p-value < 0.05. **=p-value < 0.001. Multivariable model: Wald Chi^2^=500 Degrees of freedom=16. p < 0.0001.
